# Comparison of different assays for the detection of anticyclic citrullinated peptide antibodies in patients with rheumatoid arthritis

**DOI:** 10.3389/fimmu.2022.940713

**Published:** 2022-08-02

**Authors:** Lisha Ma, Wensheng Wang, Lisha Li, Ying Chen, Binxuan Chen, Miaoli Shao, Yongjun Cheng, Renfang Zhou

**Affiliations:** ^1^ Department of Clinical Laboratory, The First People’s Hospital of Wenling, Affiliated to Wenzhou Medical University, Taizhou, China; ^2^ Department of Rheumatology, The First People’s Hospital of Wenling, Affiliated to Wenzhou Medical University, Taizhou, China

**Keywords:** anti-cyclic citrullinated peptide antibodies, diagnosis, rheumatoid arthritis, immunoturbidimetric assay, electrochemiluminescence immunoassay, chemiluminescence immunoassay

## Abstract

**Objective:**

To evaluate a novel fully automated immunoturbidimetric assay developed by Qiangsheng Biotechnology Company for the detection of anticyclic citrullinated peptide antibodies (anti-CCP) in serum of patients with rheumatoid arthritis (RA) and compare it to the conventional EUROIMMUN- anti-CCP ELISA. Two other commonly used automated assays, the Elecsys anti-CCP assay, an ECLIA that is run on the Modular Analystics E170 (Cobas Diagnostics, Germany), and an anti-CCP CLIA developed by YHLO that is run on the iFlash 3000 Chemiluminescence Immunoassay Analyzer, were included as reference standards.

**Methods:**

A total of 264 serum samples were collected from patients attending the First People’s Hospital of Wenling affiliated to Wenzhou Medical University between July 2020 and November 2020. These included 131 serum samples collected from patients with RA, 70 serum samples collected from patients with other autoimmune diseases, and 63 serum samples collected from healthy controls at a physical examination. The clinical performance and sensitivity and specificity of the four anti-CCP assays for the diagnosis of RA were compared using receiver operating characteristic (ROC) curve analysis.

**Results:**

The Kappa statistic indicated almost perfect agreement between the EUROIMMUN-anti-CCP ELISA and the Elecsys anti-CCP ECLIA (Cobas) (0.863), the EUROIMMUN-anti-CCP ELISA and the anti-CCP CLIA (YHLO) (0.862), and the Elecsys anti-CCP ECLIA (Cobas) and the anti-CCP CLIA (YHLO) (0.816). On ROC curve analysis, AUC values were 0.955 for the EUROIMMUN-anti-CCP ELISA, 0.948 for the anti-CCP CLIA (YHLO), 0.947 for the Elecsys anti-CCP ECLIA (Cobas) and 0.903 for Qiangsheng, indicating all the assays had a good diagnostic performance for RA.

**Conclusion:**

The anti-CCP assays provided similar diagnostic information. The novel fully automated immunoturbidimetric assay for anti-CCP developed by Qiangsheng Biotechnology Company may be especially useful for large scale clinical screening in RA as it has a shorter testing time than the commercially available alternatives.

## Introduction

Rheumatoid arthritis (RA) is a chronic progressive systemic autoimmune disease with a heterogeneous phenotype that ranges from mild to severe (1). In 1858, a British physician A.B. Garrod introduced the term RA, and described it as a disease of the whole joint. Modern literature indicates RA manifests as inflammation of synovial tissue, progressive destruction of cartilage, ligaments and bone, joint deformity and dysfunction, and systemic symptoms (2). RA predominantly affects women and is mostly diagnosed in individuals aged 40-60 years (3).

Anticyclic citrullinated peptide antibodies (anti-CCP) have utility for establishing a diagnosis of RA as they have excellent specificity for early RA and are markers of erosive arthritis (4). The specificity and sensitivity of anti-CCP for the diagnosis of RA are 95%–99% and 60%–75%, respectively (5, 6). In 2010, the American College of Rheumatology/European League Against Rheumatism (ACR/EULAR) identified anti-CCP as an immunologic marker of RA (7). The third generation anti-CCP enzyme-linked immunosorbent assay (ELISA) has increased sensitivity for the detection of patients with RA compared to first- and second-generation tests (8). ELISA has a diagnostic advantage for many diseases, including RA; however, it is difficult to automate because of long assay time and complex liquid handling procedures (9).

Other commercial immunoassays that detect anti-CCP include immunoturbidimetric assays, electrochemiluminescence immunoassays (ECLIAs), and chemiluminescence immunoassays (CLIAs). These are easier to automate than ELISA and affordable for clinical laboratories.

The objective of this study was to evaluate a novel fully automated immunoturbidimetric assay developed by Qiangsheng (Zhejiang Kings Biotechnology Co., Ltd.) for the detection of anti-CCP in serum of patients with RA and compare it to the conventional EUROIMMUN-anti-CCP ELISA. Two other commonly used automated assays, the Elecsys anti-CCP assay, an ECLIA that is run on the Modular Analystics E170 (Cobas Diagnostics, Germany), and an anti-CCP CLIA developed by YHLO that is run on the iFlash 3000 Chemiluminescence Immunoassay Analyzer, were included as reference standards.

## Methods

### Samples

This study used serum samples collected from patients with RA, other autoimmune diseases or healthy controls attending the First People’s Hospital of Wenling affiliated to Wenzhou Medical University between July 2020 and November 2020. Serum was obtained after routine blood testing ordered by physicians. Serum was stored at -70°C until analysis.

Use of patient data and sample collection was approved by the hospital’s institutional ethics committee. Diagnostic evaluation of anti-CCP tests did not require patient written informed consent.

### Anti-CCP antibody assays

The following anti-CCP assays were evaluated (1): The commercially available EUROIMMUN-anti-CCP ELISA (Euroimmun Medizinische Labordiagnostika AG, Germany) (2), a new immunoturbidimetric assay developed by Qiangsheng Biotechnology Company (Zhejiang Kings Biotechnology Co., Ltd.) that is run on the AU5800 clinical chemistry analyzer (Backman Coulter Inc., USA) (3); the Elecsys anti-CCP ECLIA that is run on the Modular Analystics E170 (Cobas Diagnostics, Germany), and (4) the anti-CCP CLIA that is run on the iFlash 3000-A Chemiluminescence Analyzer (Shenzhen YHLO Biotech Co., Ltd.) (**Table 1**). Anti-CCP positivity was determined according to the manufacturers’ recommended thresholds.

**Table 1:** Characteristics of anti-CCP assays.

	EUROIMMUN-anti-CCP ELISA	Qiangsheng	Elecsys anti-CCP ECLIA (Cobas)	Anti-CCP CLIA (YHLO)
**Principle of the assay**	ELISA	Immunoturbidimetric	ECLIA	CLIA
**Citrullinated antigen**	Second-generation synthetic CCPs	Third-generation CCPs	Second-generation synthetic CCPs	Third-generation synthetic CCPs
**Conjugate**	HRP-rabbit anti-human IgG	–	Ru(bpy)^2+^_3_-mouse monoclonal anti-human IgG	Acridinium-ester-labeled anti-human IgG
**Incubation time (min)**	120	5	18	35
**Sample dilution**	1:101	–	–	–
**No. of calibrators**	5	2	2	3
**Range of detection (U/mL)**	0-200	5-100	7-500	1-100
**Manufacturer’s cut-off (U/mL)**	5	35	17	5

### Clinical diagnosis

Clinical diagnoses were made based on a retrospective review of patients’ medical records. RA was diagnosed according to the 2010 ACR/EULAR classification criteria (7). Sjögren’s syndrome (SS) was diagnosed according to the ACR/EULAR classification criteria (10). Systemic lupus erythematosus (SLE) was diagnosed according to the ACR criteria (11). Connective tissue disease (CTD) was defined according to the most recent criteria from the Japanese Research Committee of the Ministry of Health, Labor, and Welfare (12). Ankylosing spondylitis (AS) was diagnosed according to the Rome and New York criteria (13). Systemic scleroderma (SSc) was diagnosed according to the 2013 ACR/EULAR classification criteria (14).

### Statistical analysis

Statistical analyses were performed using SPSS for Windows (version 15; SPSS Inc., Chicago, IL, USA), GraphPad Prism 5 (GraphPad Software Inc, USA), R software (version 3.6.2, https://www.r-project.org/) and the Deepwise & Beckman Coulter DxAI platform (https://dxonline.deepwise.com ). Qualitative agreement between the four assays was evaluated with Kappa statistics (15). The kappa coefficient was positive if the observed agreement exceeded the agreement expected by chance. The kappa coefficient was negative if the observed agreement was less than the agreement expected by chance. Clinical performance and sensitivity and specificity of the assays for the diagnosis of RA were compared using receiver operating characteristic (ROC) curve analysis.

## Results

A total of 264 serum samples were collected from patients attending the First People’s Hospital of Wenling affiliated to Wenzhou Medical University between July 2020 and November 2020. These included 131 serum samples collected from patients with RA, 70 serum samples collected from patients with other autoimmune diseases (ANCA-associated vasculitis [AAV], n=5; SS, n=21; CTD, n=5; AS, n=9; SLE, n=24; SSc, n=6), and 63 serum samples collected from healthy controls at a physical examination.

Median age of patients with RA, other autoimmune diseases, and healthy controls were 57.1 ± 12.4 years, 46.8 ± 15.1 years, and 38.0 ± 9.0 years, respectively. The male: female ratio in patients with RA, other autoimmune diseases, and healthy controls was 27:104, 1:6 and 3:4, respectively. There were no significant differences in age or male: female ratio between groups.

Among RA patients, anti-CCP positivity was highest with the Elecsys anti-CCP ECLIA (Cobas) (90.1%) and Qiangsheng (90.1%), followed by the EUROIMMUN-anti-CCP ELISA (87%) and the anti-CCP CLIA (YHLO) (84.7%). Among patients with other autoimmune diseases, anti-CCP positivity was highest with Qiangsheng (42.9%), followed by the EUROIMMUN-anti-CCP ELISA (5.7%) and the Elecsys anti-CCP ECLIA (Cobas) (5.7%), and the anti-CCP CLIA (YHLO) (4.3%). Among healthy controls, anti-CCP positivity was highest with Qiangsheng (6.3%), followed by the anti-CCP CLIA (YHLO) (3.2%). No healthy controls were positive on the EUROIMMUN-anti-CCP ELISA or the Elecsys anti-CCP ECLIA (Cobas) (**Table 2**).

**Table 2:** Patient demographics and anti-CCP positivity.

Control (n=133)			
	RA (n=131)	Other autoimmune disease (n=70)	Healthy control (n=63)
**Age, year median**	57.099 ± 12.376	46.829 ± 15.099	38.016 ± 8.951
**Male : Female n (%)**	27 (20.6),104 (79.4)	10 (14.3), 60 (85.7)	27 (42.9), 36 (57.1)
**EUROIMMUN-Anti-CCP ELISA, n (%)**	114 (87.0)	4 (5.7)	0 (0)
**Qiangsheng, n (%)**	118 (90.1)*	30 (42.9)*	4 (6.3)*
**Elecsys anti-CCP ECLIA (Cobas), n (%)**	118 (90.1)*	4 (5.7)#	0 (0)#
**Anti-CCP CLIA (YHLO), n (%)**	111 (84.7)#	3 (4.3)#	2 (3.2)#

^*^P<0.05, ^#^P >0.05: other anti-CCP assays vs. ELISA; Fisher exact test

### Probability of agreement

The Kappa statistic indicated almost perfect agreement between the EUROIMMUN-anti-CCP ELISA and the Elecsys anti-CCP ECLIA (Cobas) (0.863), the EUROIMMUN-anti-CCP ELISA and the anti-CCP CLIA (YHLO) (0.862), and the Elecsys anti-CCP ECLIA (Cobas) and the anti-CCP CLIA (YHLO) (0.816). There was moderate/weak agreement between Qiangsheng and the anti-CCP ECLIA (Cobas) (0.655), Qiangsheng and the anti-CCP CLIA (YHLO) (0.598), and Qiangsheng and the EUROIMMUN-anti-CCP ELISA (0.582) (**Table 3**) (15).

**Table 3:** Kappa statistic.

Assay	EUROIMMUN-anti-CCP ELISA	Qiangsheng	Elecsys anti-CCP ECLIA (Cobas)
**Qiangsheng** **Elecsys anti-CCP ECLIA (Cobas)** **Anti-CCP CLIA (YHLO)**	0.582 0.863 0.862	- 0.655 0.598	- - 0.816

Probability of agreement [cite: McHugh ML. Interrater reliability: the kappa statistic. Biochem Med (Zagreb). 2012;22 (3):276-282.]

0.4-0.59, weak

0.60-0.79, moderate

0.80-0.90, strong

> 0.90, almost perfect

**Figure 1:**
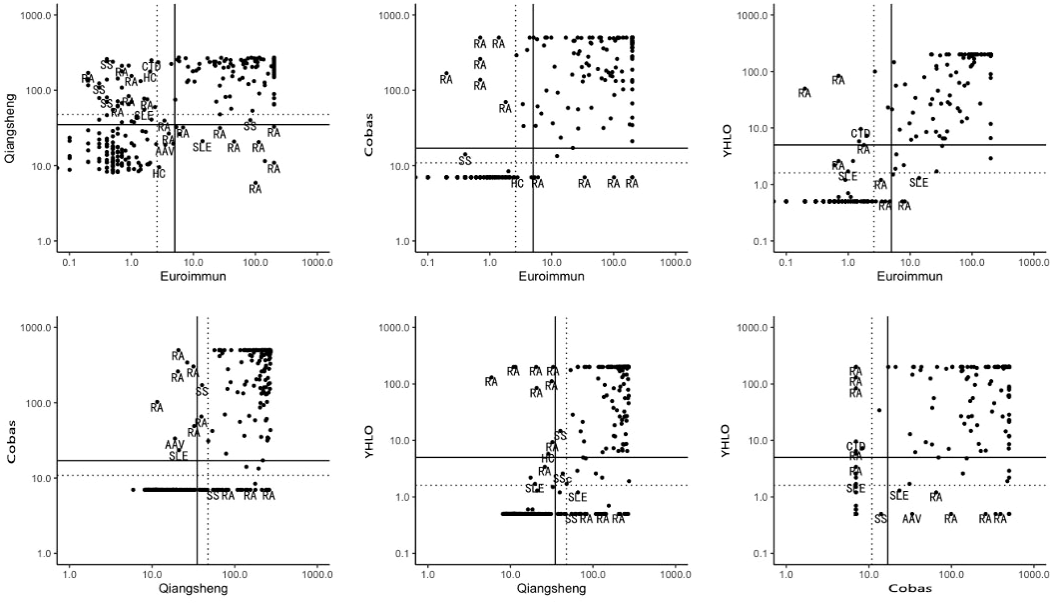
Discrepant results between assays and their clinical diagnoses. Solid lines: manufacturers’ cut-off values; broken lines: optimal cut-off. RA, rheumatoid arthritis; SLE, systemic lupus erythematosus; SS, Sjögren’s syndrome; CTD, connective tissue disease; SSc, systemic scleroderma; HC, healthy person; AAV, ANCA-associated vasculitis.

Discrepant results between the assays and their clinical diagnoses are shown in **Figure 1**.

### Diagnostic performance

**Figure 2:**
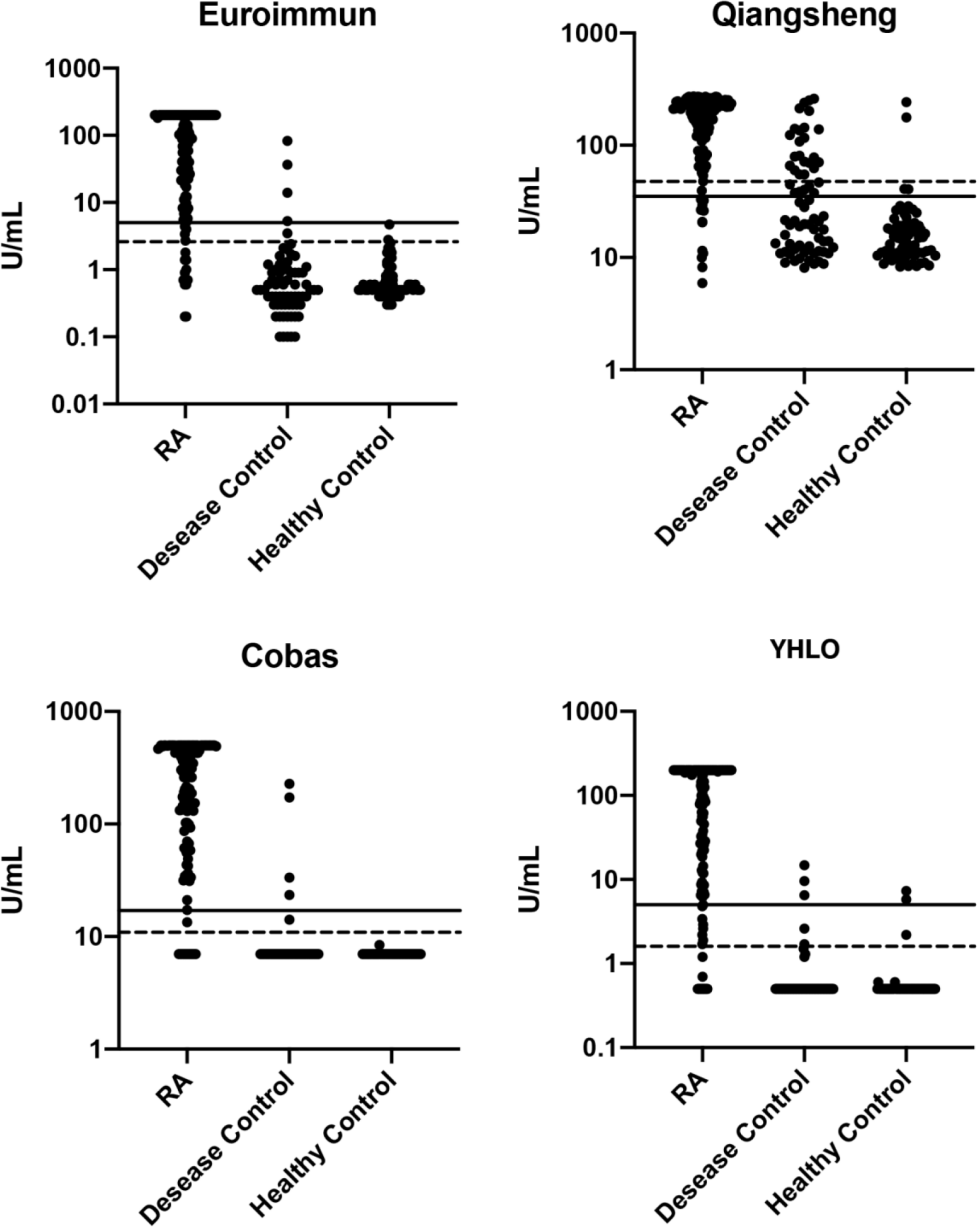
Scatter plots of anti-CCP levels (log-scale) in patients with RA, other autoimmune disease, and healthy controls. Solid lines: manufacturers’ cut-off values; broken lines: optimal cut-off. RA, rheumatoid arthritis.

Scatter plots of anti-CCP levels in patients with RA, other autoimmune diseases, and healthy controls on the four assays are shown in **Figure 2**. The sensitivity and specificity of the assays for the diagnosis of RA are presented in **Table 4**. At the cut-offs proposed by the manufacturers, assay sensitivities ranged from 84.7% to 90.1%, and specificities ranged from 74.4% to 97.0%.

**Table 4:** Diagnostic performance.

	Cut-off level (U/mL)	Sensitivity (%)	Specificity (%)
**EUROIMMUN-anti-CCP ELISA**	5.0^*^	87.0	97.0
	2.6^#^	90.1	94.7
**Qiangsheng**	35.0^*^	90.1	74.4
	47.5^#^	89.3	81.2
**Elecsys anti-CCP ECLIA (Cobas)**	17.0^*^	90.1	97.0
	10.9^#^	90.8	96.2
**Anti-CCP CLIA (YHLO)**	5.0^*^	84.7	96.2
	1.6^#^	90.1	94.0

*Manufacturer’s cut-off; #Optimal cut-off obtained by receiver operating characteristic (ROC) curve analysis.

**Figure 3:**
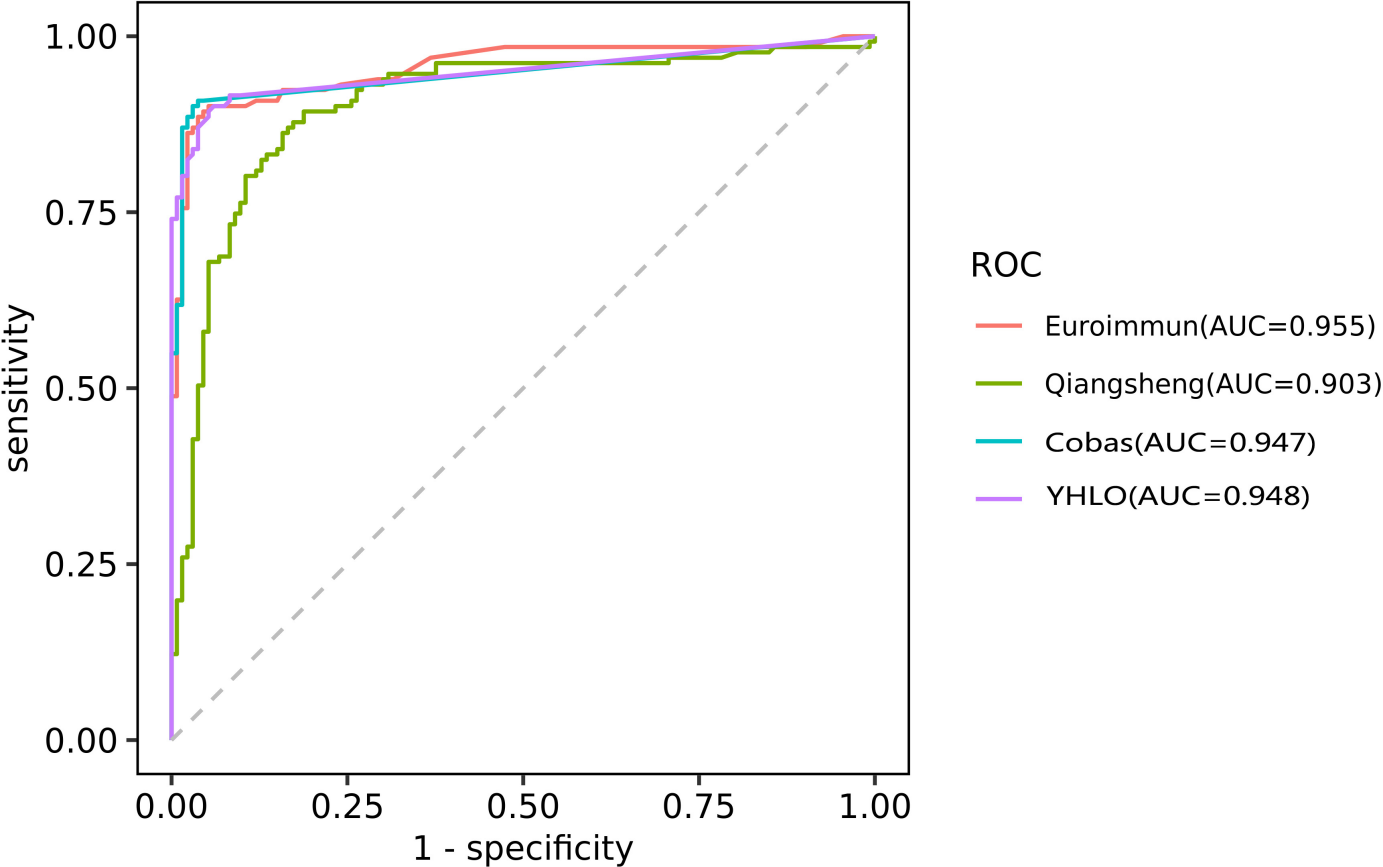
ROC curve analysis evaluating the diagnostic performance of the EUROIMMUN-anti-CCP ELISA, Qiangsheng, Elecsys anti-CCP ECLIA (Cobas) and the anti-CCP CLIA (YHLO) for RA.AUC as a measure of diagnostic performance: [citation: Mandrekar JN. Receiver operating characteristic curve in diagnostic test assessment. J Thorac Oncol. 2010;5 (9):1315-1316. doi:10.1097/JTO.0b013e3181ec173d] 0.5, no discrimination 0.7-0.8, acceptable 0.8-0.9, excellent >0.9, outstanding.

ROC curve analysis was used to further evaluate the diagnostic performance of the assays for RA (**Figure 3**). The AUC values were 0.955 for the EUROIMMUN-anti-CCP ELISA, 0.948 for the anti-CCP CLIA (YHLO), 0.947 for the Elecsys anti-CCP ECLIA (Cobas) and 0.903 for Qiangsheng, indicating that all the assays had good diagnostic performance for RA. At the optimal cut-offs determined by ROC curve analysis, assay sensitivities ranged from 89.3% to 90.8%, and specificities ranged from 94.0% to 97.0%.

## Discussion

Rheumatoid factor (RF) is an autoantibody that has been used in the diagnosis of RA; however, RF is not specific to RA and can be affected by many factors. Accumulating evidence suggests increased protein citrullination is linked to RA, and anti-CCP positivity has greater sensitivity and specificity than RF for the diagnosis of RA (16–19). In particular, anti-CCP antibodies are valuable in the early diagnosis of RA and are an important predictor of progression and radiological damage (18, 20).

At present, ELISA is commonly used to detect anti-CCP antibodies for RA diagnosis. However, ELISA can be time-consuming as it involves binding an antibody in the liquid phase to a target antigen attached to a solid phase and identifying bound antibody by generating and detecting a signal (21). Automation is essential, especially in high-volume laboratories.

This study evaluated the utility of four anti-CCP assays as diagnostic tools in RA. These included an ELISA, ECLIA and CLIA, which are commonly used in clinical laboratories, as well as a novel fully automated immunoturbidimetric assay for anti-CCP developed by Qiangsheng Biotechnology Company. The immunoturbidimetric assay format comprises CCP adsorbed onto latex particles, which clump in the presence of specific antibody. The concentration of anti-CCP antibody is estimated from the change in turbidity of the test sample using an AU5800 clinical chemistry analyzer. The Qiangsheng immunoturbidimetric assay provided results in 5 minutes, while the ELISA, ECLIA and CLIA required 18 to 120 minutes.

Among the RA patients included in this study, anti-CCP positivity was highest with the Elecsys anti-CCP ECLIA (Cobas) (90.1%) and Qiangsheng (90.1%). However, Qiangsheng had low specificity for RA, as anti-CCP positivity was 42.9% among patients with other autoimmune diseases and 6.3% among healthy controls. The Kappa statistic indicated moderate/weak agreement between Qiangsheng and the anti-CCP ECLIA (Cobas) (0.655), the anti-CCP CLIA (YHLO) (0.598), and the EUROIMMUN-anti-CCP ELISA (0.582) (15). ROC curve analysis suggested all the assays evaluated in this study had good diagnostic performance for RA (22).

Discrepant results between the assays and their clinical diagnoses may have arisen for a number of reasons. First, autoantibodies in patients with RA are heterogeneous, having different affinities, subtypes, and glycosylation patterns. Second, technical aspects of the assays varied, including cut-off values, methods of calibration and quantitation, choice of solid phase and coating, and type and source of antigen (23). Last, the performance of different detection methods may be influenced by antigen epitopes (linear epitopes, spatial conformation epitopes).

The newly developed Qiangsheng assay has several advantages. It requires only 5 minutes to test a sample, automation makes the assay attractive, especially for high volume laboratories, and it can be used on the AU5800 clinical chemistry analyzer. However, the specificity of the Qiangsheng assay for RA is relatively weak compared to the other assays, especially for patients with other autoimmune diseases. This leads to a high rate of false positives. This does have some clinical benefit, as it initiates further screening of these patients, which may lead to the timely diagnosis and treatment of other automimmune diseases.

## Conclusion

Appropriate selection of commercial anti-CCP assays is essential for high volume laboratories. The present study suggested the EUROIMMUN-anti-CCP ELISA, the Elecsys anti-CCP ECLIA, the anti-CCP CLIA (YHLO), and a novel immunoturbidimetric assay developed by Qiangsheng Biotechnology Company (Qiangsheng) had a good diagnostic performance for RA. Anti-CCP positivity was determined according to the manufacturers’ recommended thresholds, providing evidence that these anti-CCP assays offer similar diagnostic information. The novel fully automated immunoturbidimetric assay for anti-CCP developed by Qiangsheng Biotechnology Company may be especially useful for large scale clinical screening in RA as it has a shorter testing time than the commercially available alternatives.

## Data availability statement

The raw data supporting the conclusions of this article will be made available by the authors, without undue reservation.

## Ethics statement

The studies involving human participants were reviewed and approved by Ethics Committee of First People’s Hospital of Wenling. Written informed consent for participation was not required for this study in accordance with the national legislation and the institutional requirements.

## Author contributions

All authors were involved in the design of this study. RZ conducted a literature search and conceived the study. LM drafted the manuscript. WW, LL, YC, and BC independently extracted and analyzed data. MS and YJC were involved in protocol development, gaining ethical approval, and data analysis. All authors reviewed and edited the manuscript and approved the final version of the manuscript.

## Funding

This work was supported by the Medical Health Science and Technology Project of Zhejiang Provincial Health Commission (2020RC148, 2022ky1413).

## Acknowledgments

We thank Medjaden Inc. for scientific editing of this manuscript.

## Conflict of interest

The authors declare that the research was conducted in the absence of any commercial or financial relationships that could be construed as a potential conflict of interest.

## Publisher's note

All claims expressed in this article are solely those of the authors and do not necessarily represent those of their affiliated organizations, or those of the publisher, the editors and the reviewers. Any product that may be evaluated in this article, or claim that may be made by its manufacturer, is not guaranteed or endorsed by the publisher.
